# Supplementary figures and images for: Analysis of CDPK1 targets identifies a trafficking adaptor complex that regulates microneme exocytosis in Toxoplasma
Source: eLife. 2023 Nov 7;12:RP85654. doi: 10.7554/eLife.85654 (PMC10629828; doi:10.7554/eLife.85654)

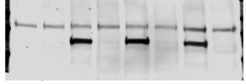

Supplement: Figure 2—source data 1. [file elife-85654-fig2-data1.zip › Figure 2-source data 1.tif]

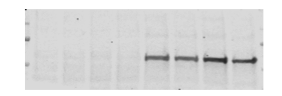

Supplement: Figure 2—source data 2. [file elife-85654-fig2-data2.zip › Figure 2-source data 2.tif]

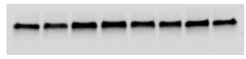

Supplement: Figure 2—source data 3. [file elife-85654-fig2-data3.zip › Figure 2-source data 3.tif]

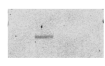

Supplement: Figure 2—source data 4. [file elife-85654-fig2-data4.zip › Figure 2-source data 4.tif]

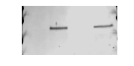

Supplement: Figure 2—source data 5. [file elife-85654-fig2-data5.zip › Figure 2-source data 5.tif]

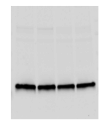

Supplement: Figure 2—source data 6. [file elife-85654-fig2-data6.zip › Figure 2-source data 6.tif]

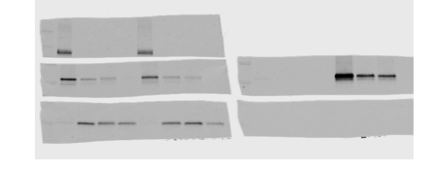

Supplement: Figure 2—source data 7. [file elife-85654-fig2-data7.zip › Figure 2-source data 7.tif]

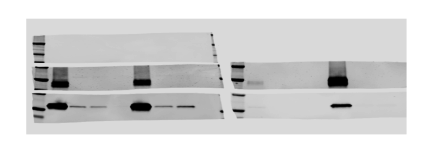

Supplement: Figure 2—source data 8. [file elife-85654-fig2-data8.zip › Figure 2-source data 8.tif]

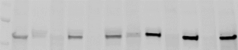

Supplement: Figure 2—source data 9. [file elife-85654-fig2-data9.zip › Figure 2-source data 9.tif]

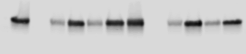

Supplement: Figure 2—source data 10. [file elife-85654-fig2-data10.zip › Figure 2-source data 10.tif]

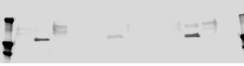

Supplement: Figure 2—source data 11. [file elife-85654-fig2-data11.zip › Figure 2-source data 11.tif]

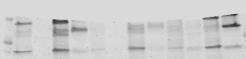

Supplement: Figure 2—source data 12. [file elife-85654-fig2-data12.zip › Figure 2-source data 12.tif]

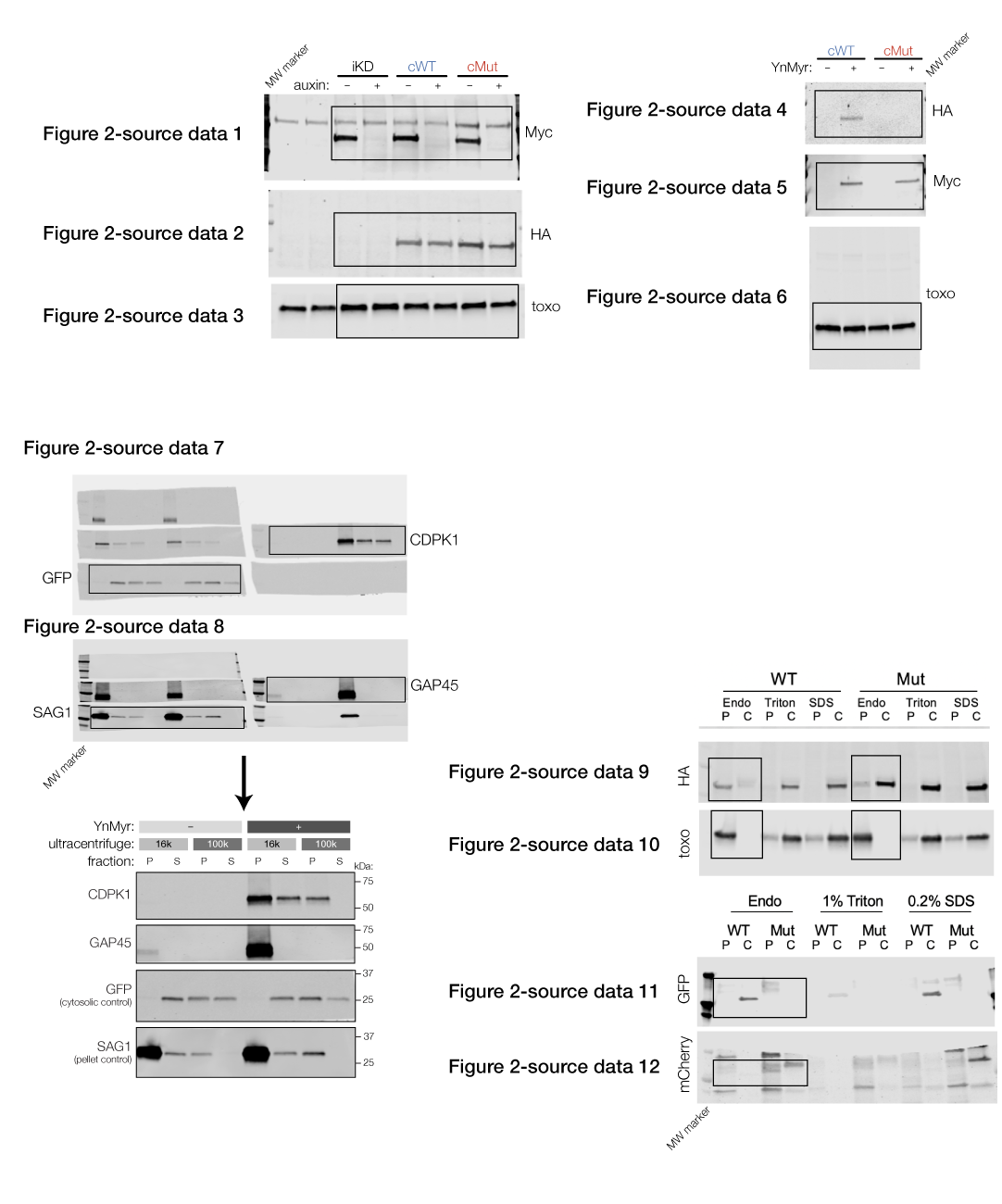

Supplement: Figure 2—source data 13. [file elife-85654-fig2-data13.zip › Figure 2-source data 13 .tif]

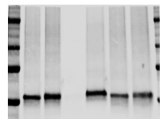

Supplement: Figure 2—figure supplement 1—source data 1. [file elife-85654-fig2-figsupp1-data1.zip › Figure 2-figure supplement 1-source data 1.tif]

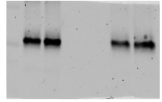

Supplement: Figure 2—figure supplement 1—source data 2. [file elife-85654-fig2-figsupp1-data2.zip › Figure 2-figure supplement 1-source data 2.tif]

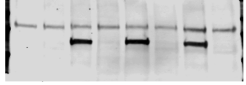

Supplement: Figure 2—figure supplement 1—source data 3. [file elife-85654-fig2-figsupp1-data3.zip › Figure 2-figure supplement 1-source data 3.tif]

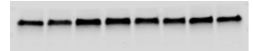

Supplement: Figure 2—figure supplement 1—source data 4. [file elife-85654-fig2-figsupp1-data4.zip › Figure 2-figure supplement 1-source data 4.tif]

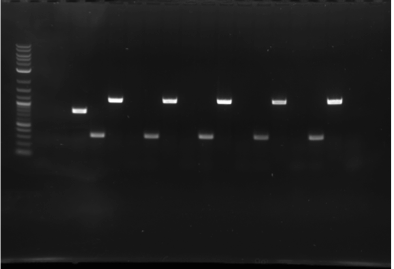

Supplement: Figure 2—figure supplement 1—source data 5. [file elife-85654-fig2-figsupp1-data5.zip › Figure 2-figure supplement 1-source data 5.tif]

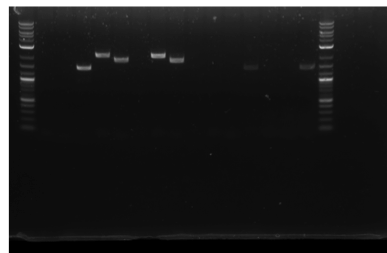

Supplement: Figure 2—figure supplement 1—source data 6. [file elife-85654-fig2-figsupp1-data6.zip › Figure 2-figure supplement 1-source data 6.tif]

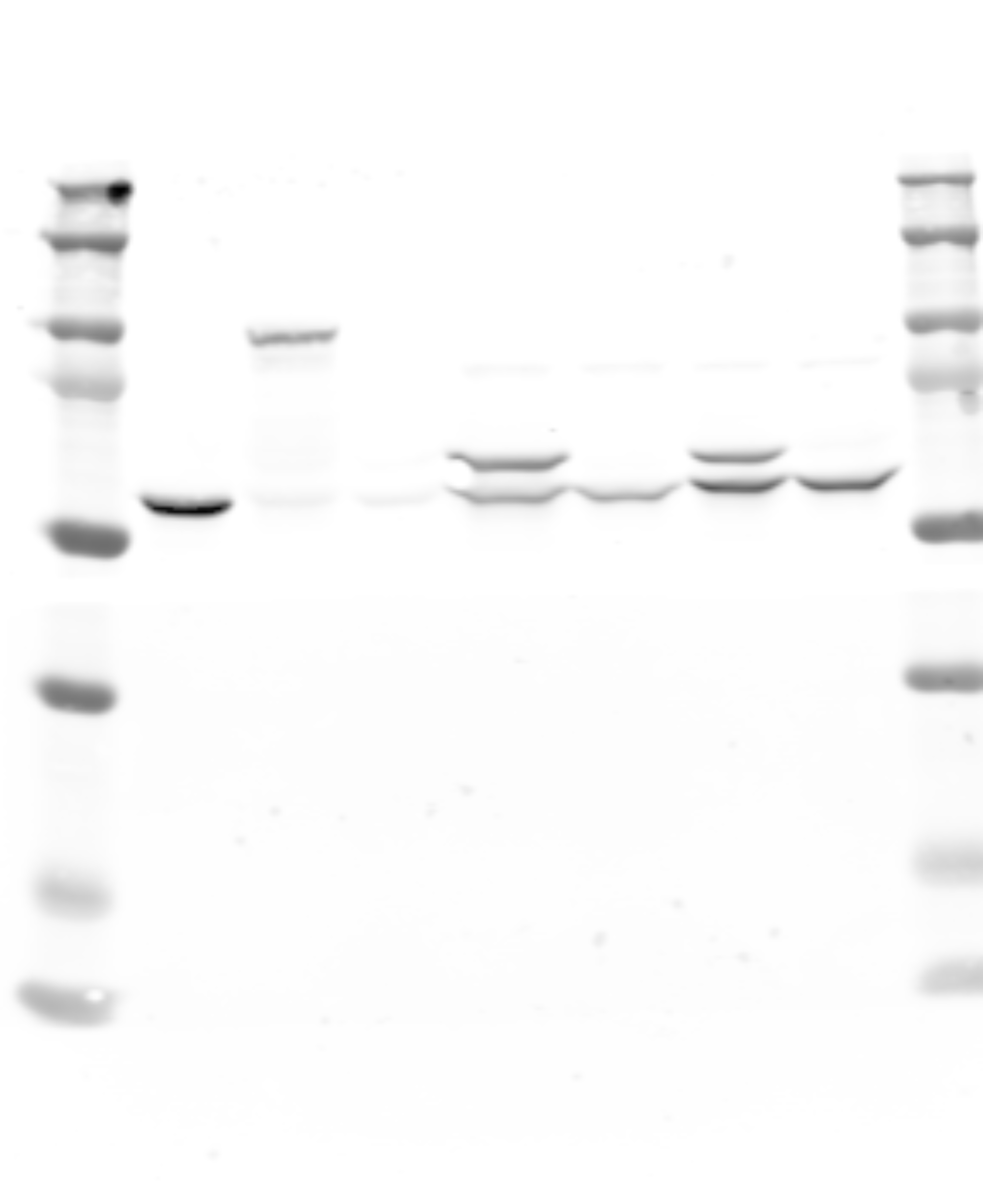

Supplement: Figure 2—figure supplement 1—source data 7. [file elife-85654-fig2-figsupp1-data7.zip › Figure 2-figure supplement 1-source data 7.tif]

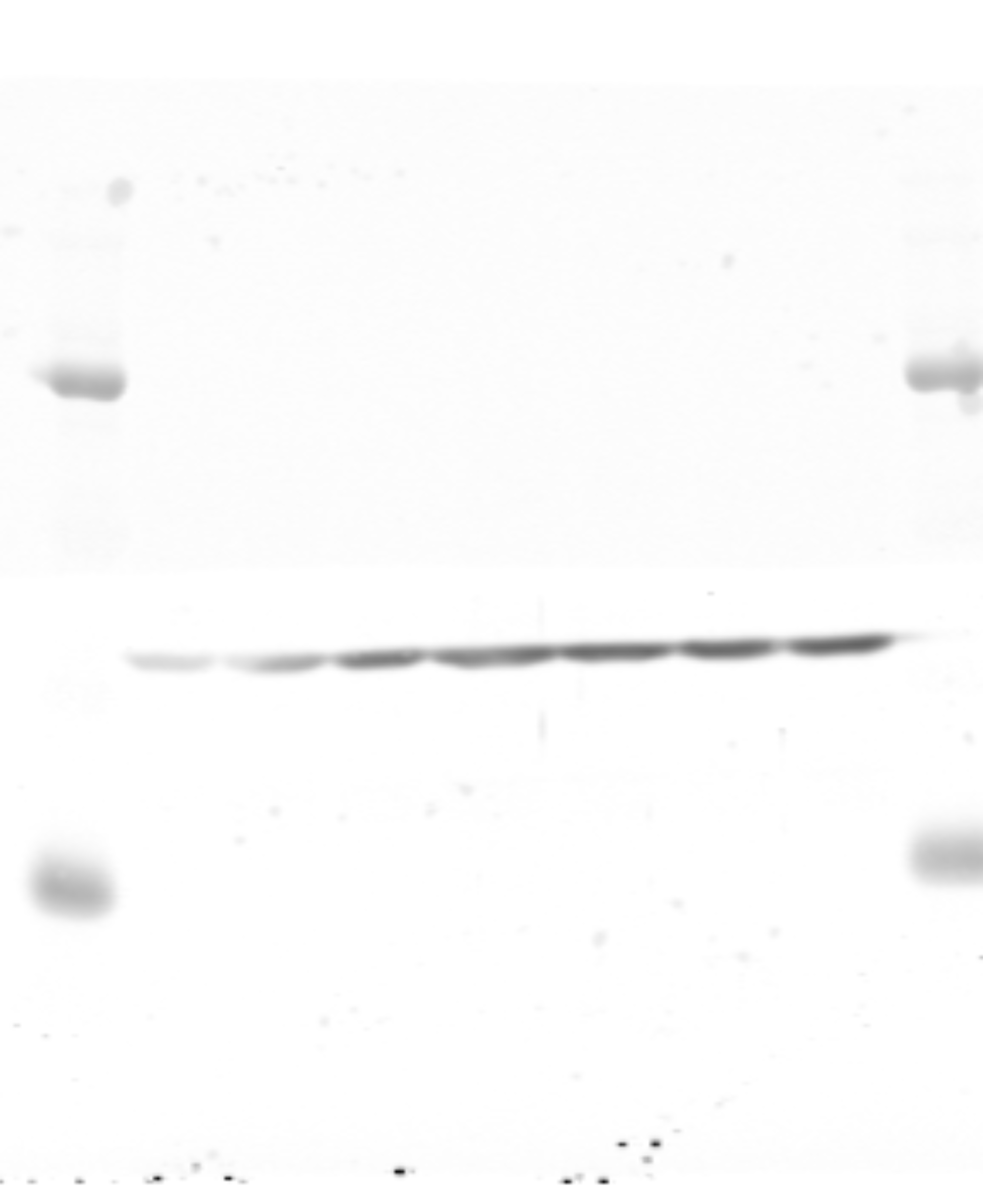

Supplement: Figure 2—figure supplement 1—source data 8. [file elife-85654-fig2-figsupp1-data8.zip › Figure 2-figure supplement 1-source data 8.tif]

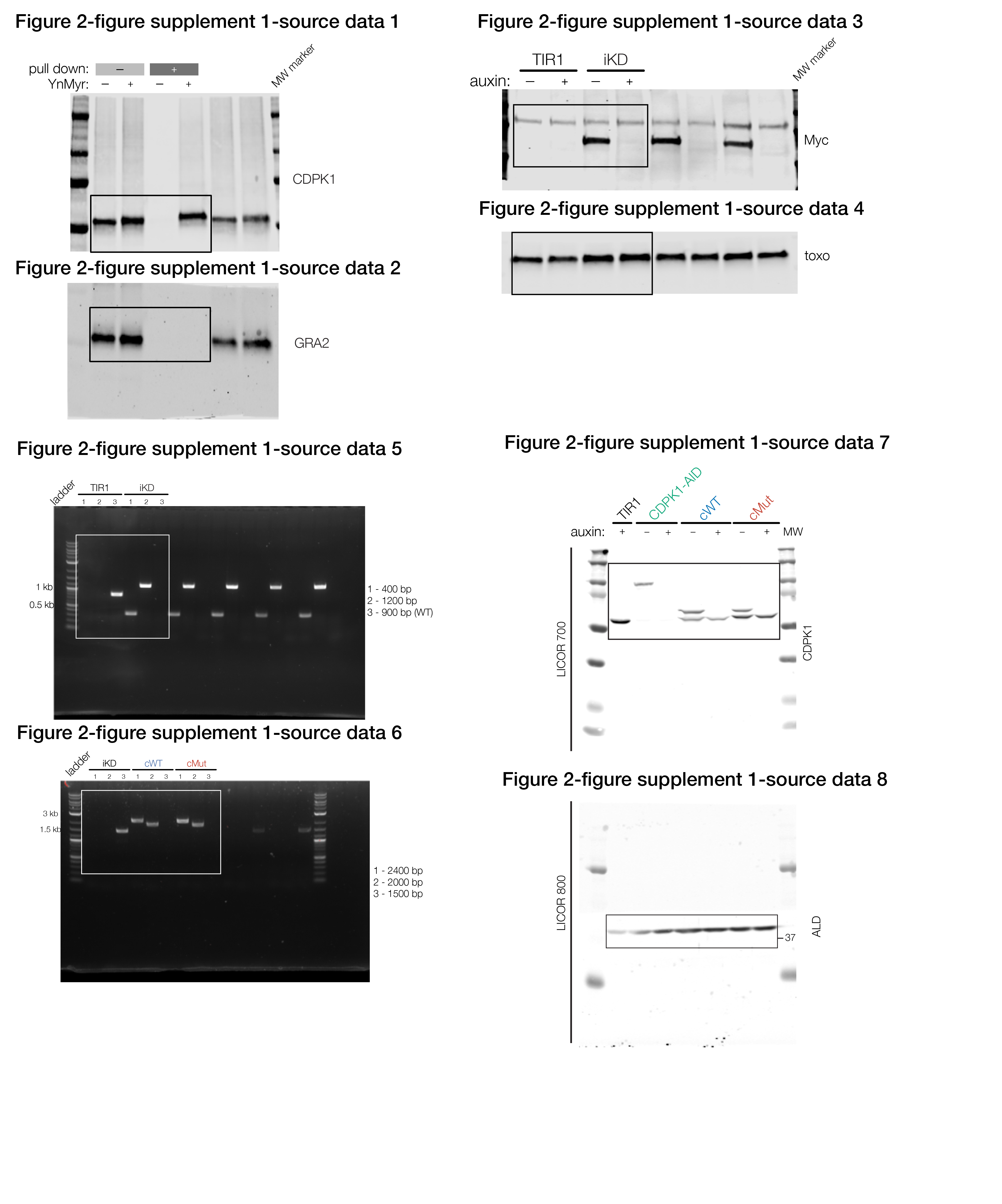

Supplement: Figure 2—figure supplement 1—source data 9. [file elife-85654-fig2-figsupp1-data9.zip › Figure 2-figure supplement 1-source data 9.png]

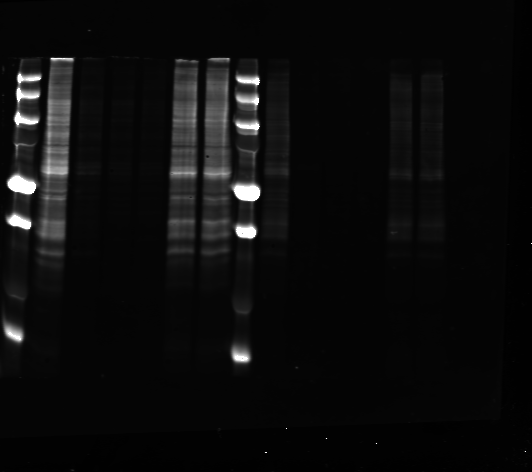

Supplement: Figure 3—source data 1. [file elife-85654-fig3-data1.zip › Figure 3-source data 1.tif]

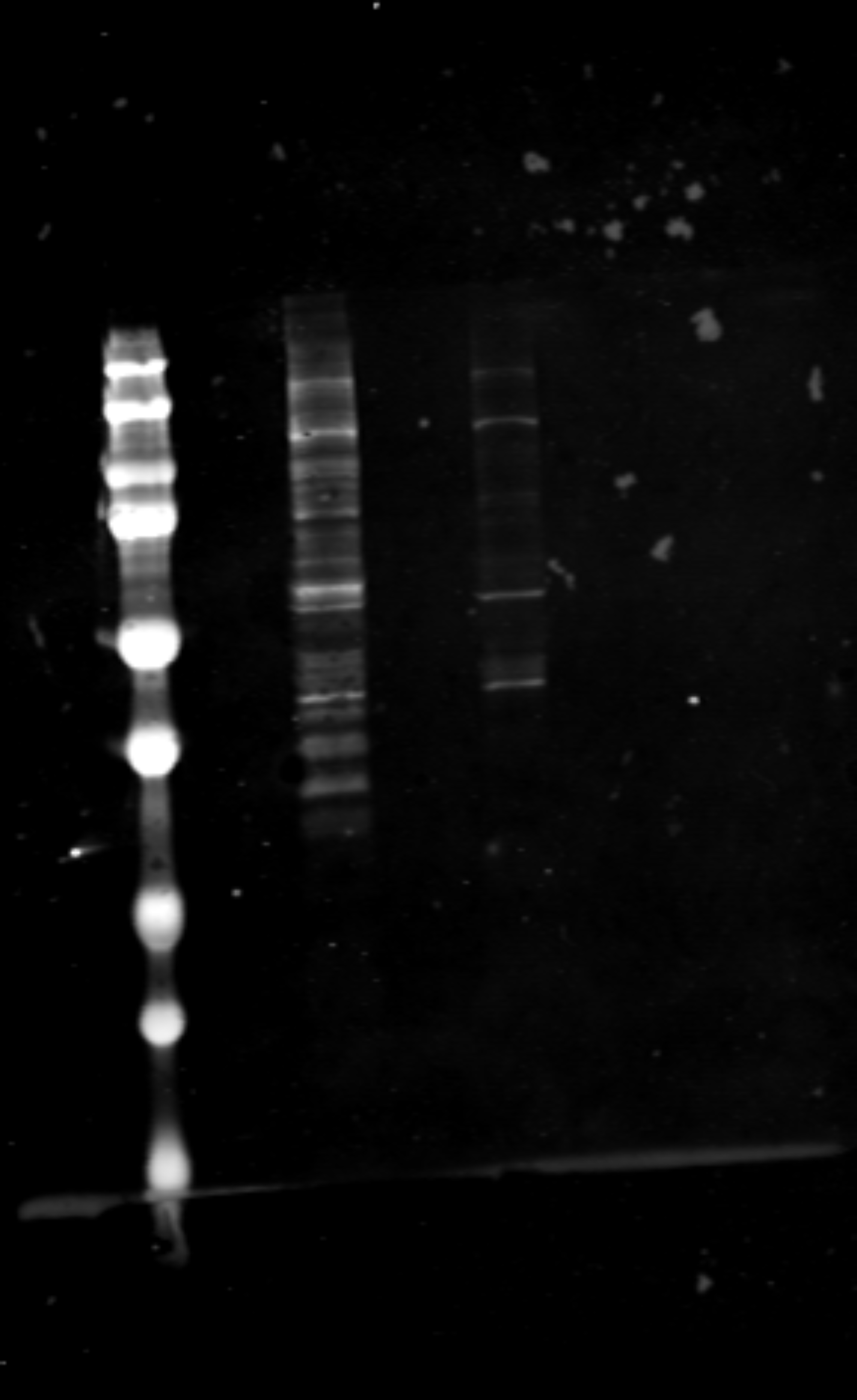

Supplement: Figure 3—source data 2. [file elife-85654-fig3-data2.zip › Figure 3-source data 2.tif]

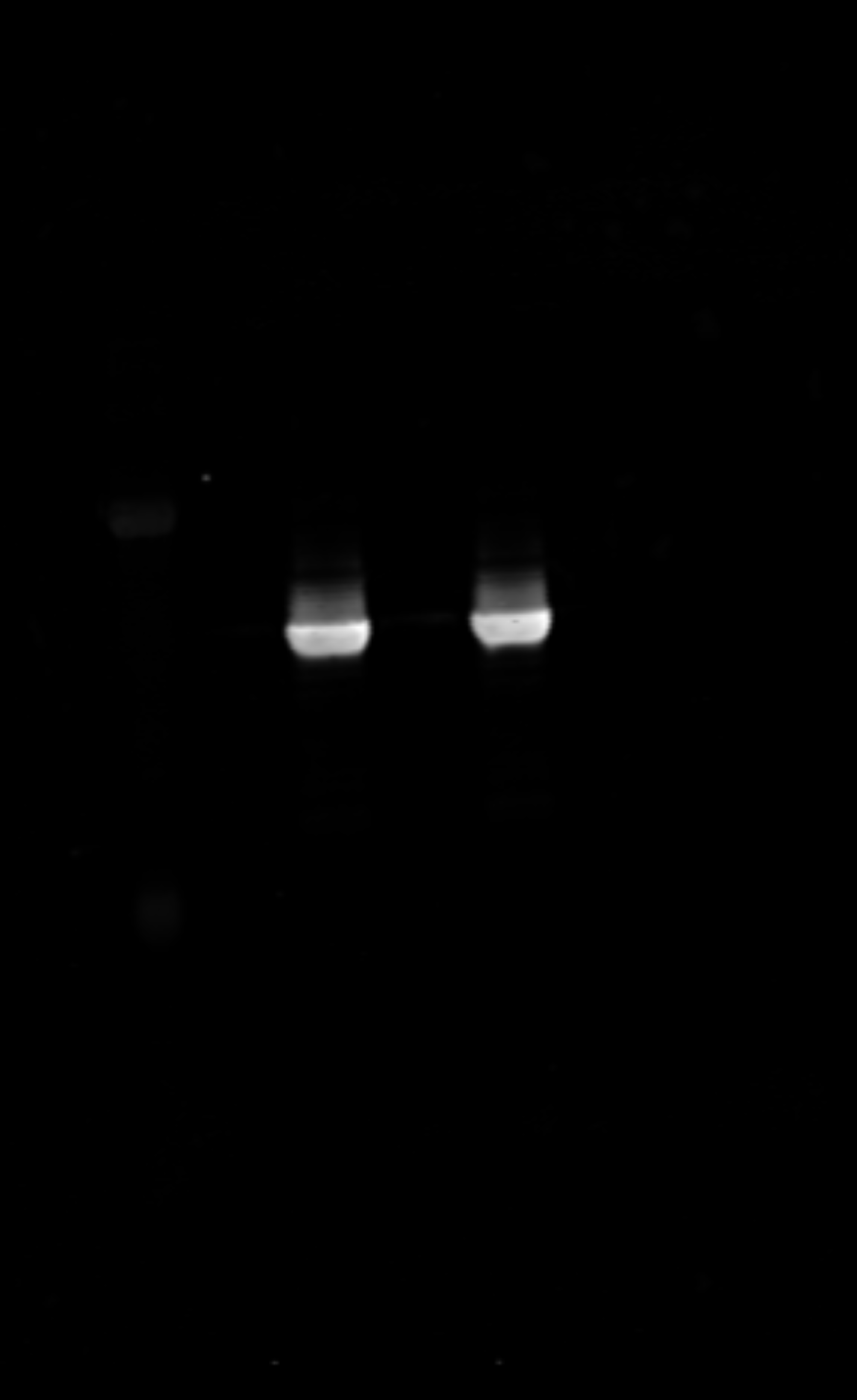

Supplement: Figure 3—source data 3. [file elife-85654-fig3-data3.zip › Figure 3-source data 3.tif]

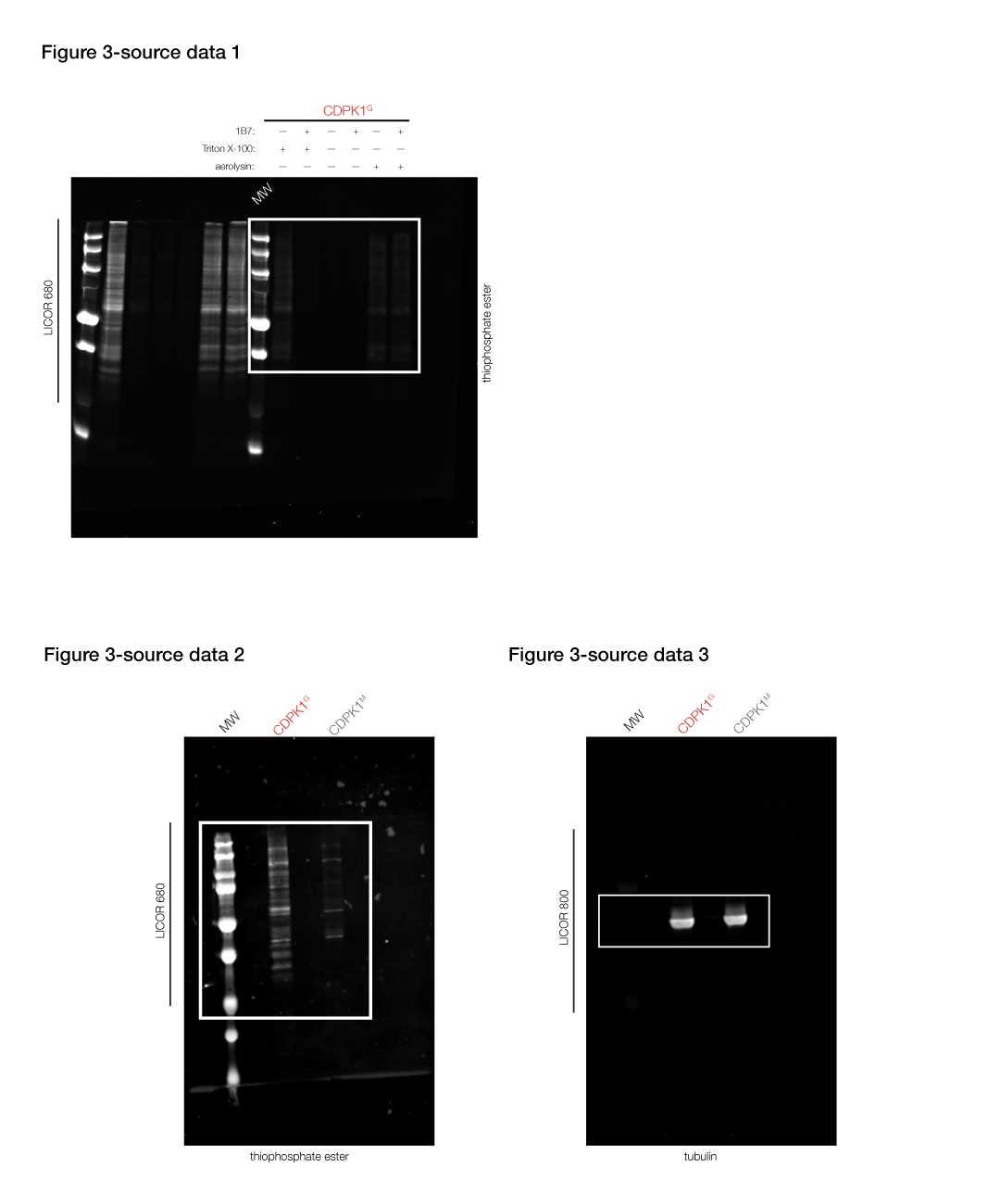

Supplement: Figure 3—source data 4. [file elife-85654-fig3-data4.zip › Figure 3-source data 4.tif]

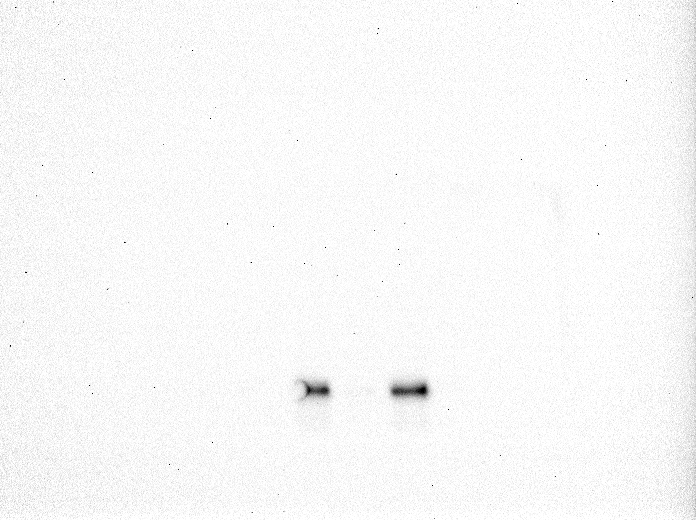

Supplement: Figure 4—source data 1. [file elife-85654-fig4-data1.zip › Figure 4-source data 1.tif]

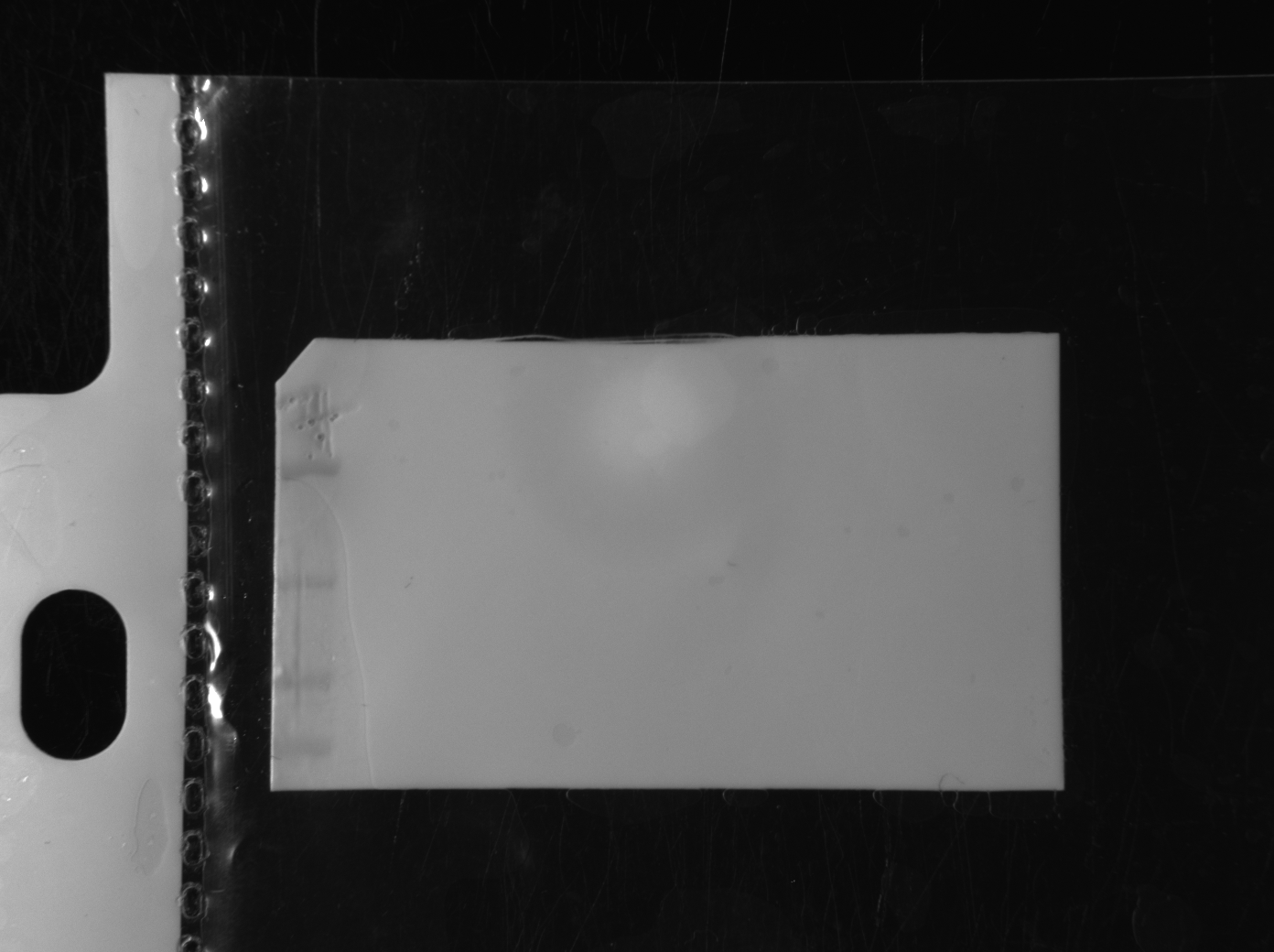

Supplement: Figure 4—source data 2. [file elife-85654-fig4-data2.zip › Figure 4-source data 2.tif]

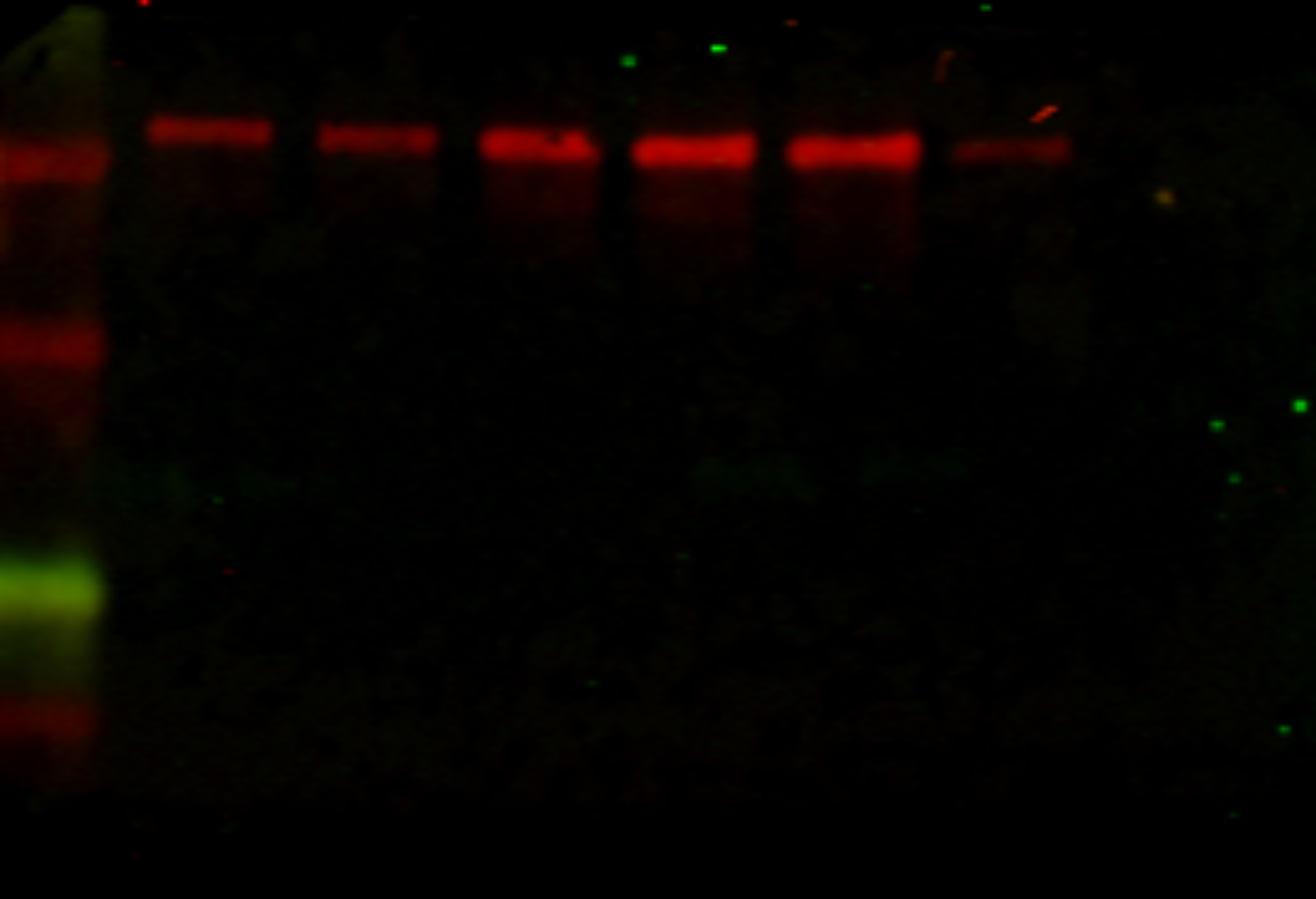

Supplement: Figure 4—source data 3. [file elife-85654-fig4-data3.zip › Figure 4-source data 3.tif]

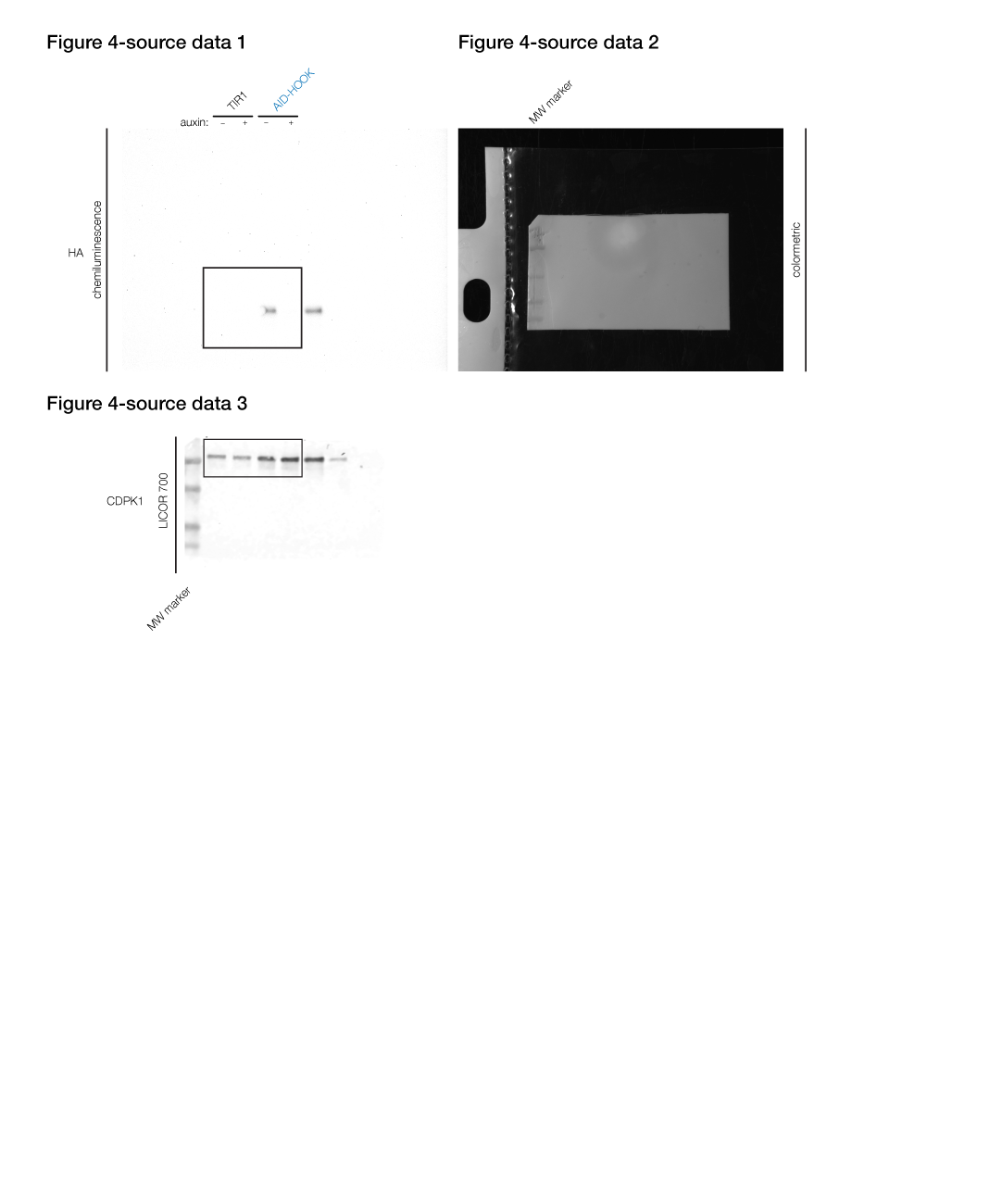

Supplement: Figure 4—source data 4. [file elife-85654-fig4-data4.zip › Figure 4-source data 4.tif]

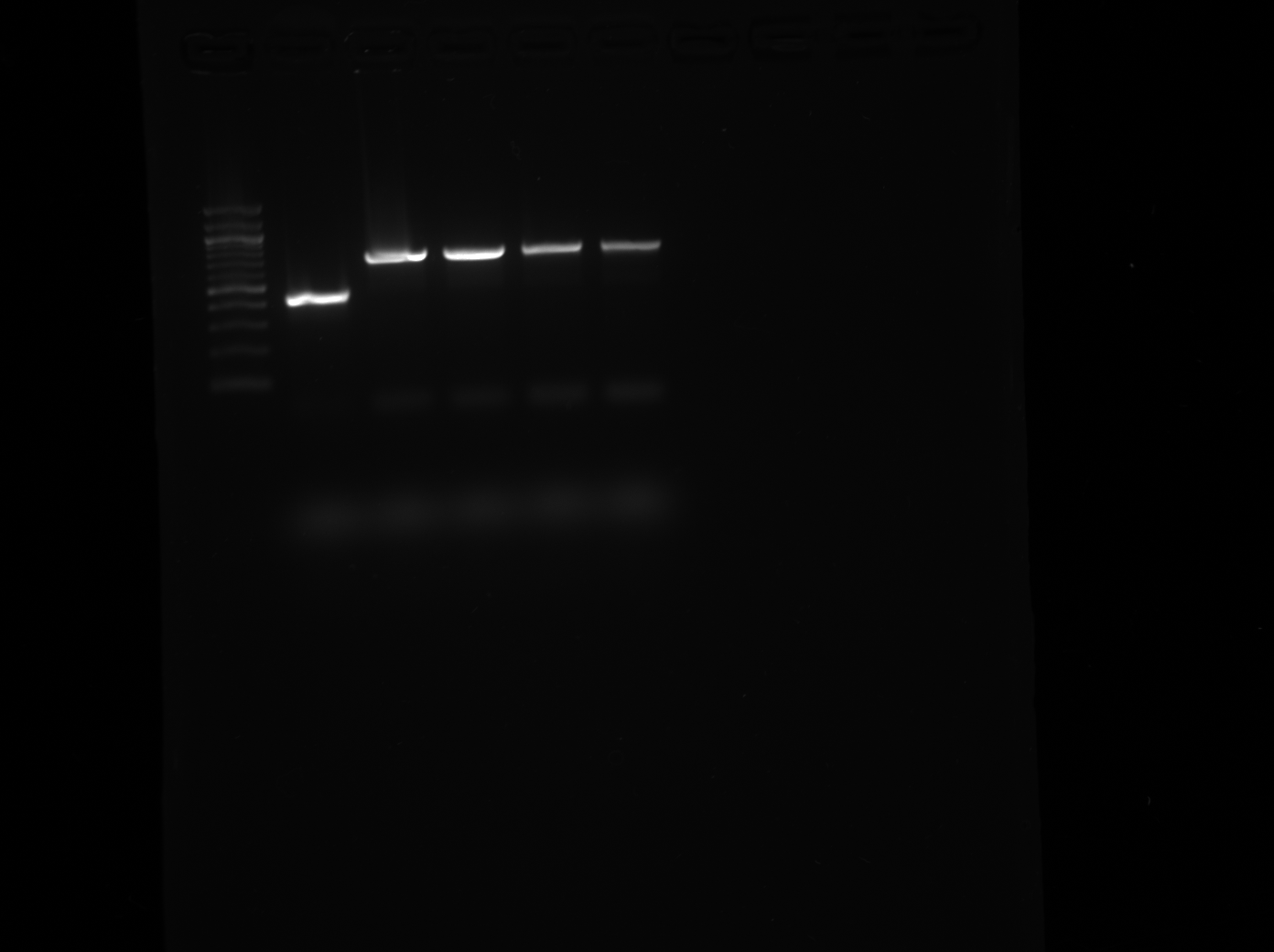

Supplement: Figure 4—figure supplement 1—source data 1. [file elife-85654-fig4-figsupp1-data1.zip › Figure 4-figure supplement 1-source data 1.tif]

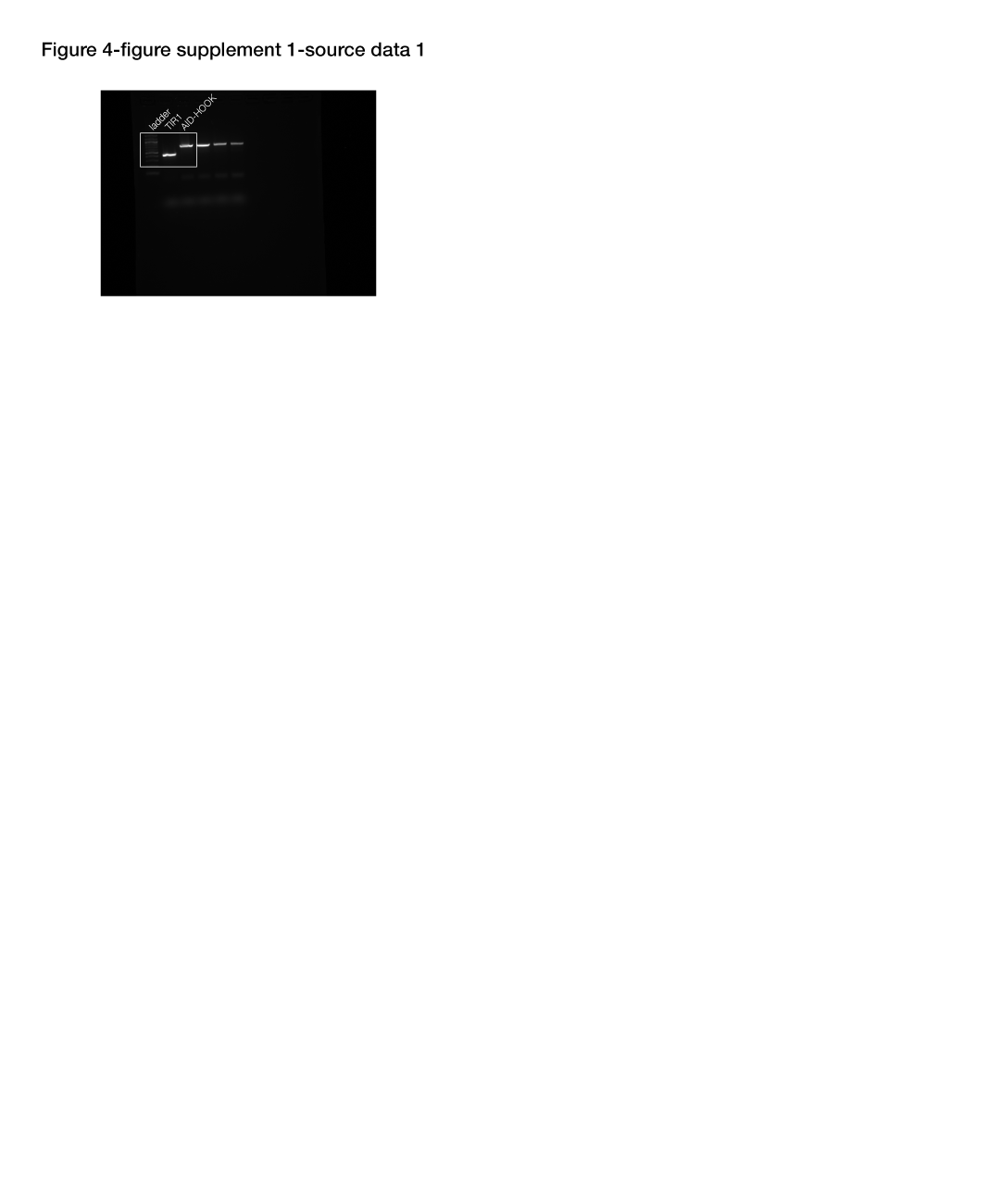

Supplement: Figure 4—figure supplement 1—source data 2. [file elife-85654-fig4-figsupp1-data2.zip › Figure 4-figure supplement 1-source data 2.tif]

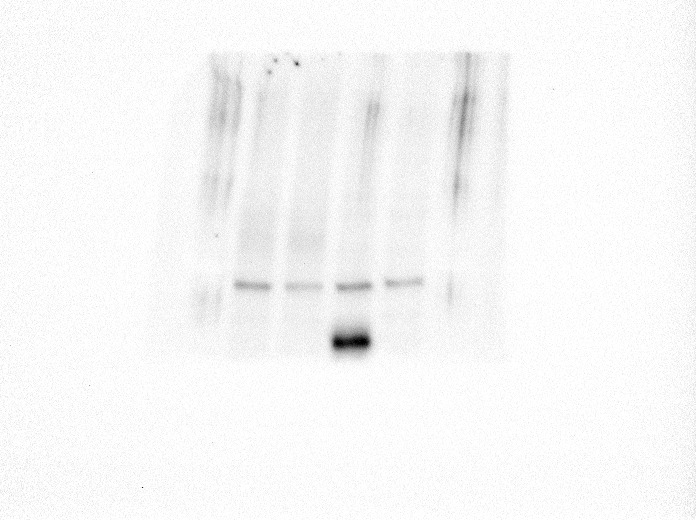

Supplement: Figure 5—source data 1. [file elife-85654-fig5-data1.zip › Figure 5-source data 1.tif]

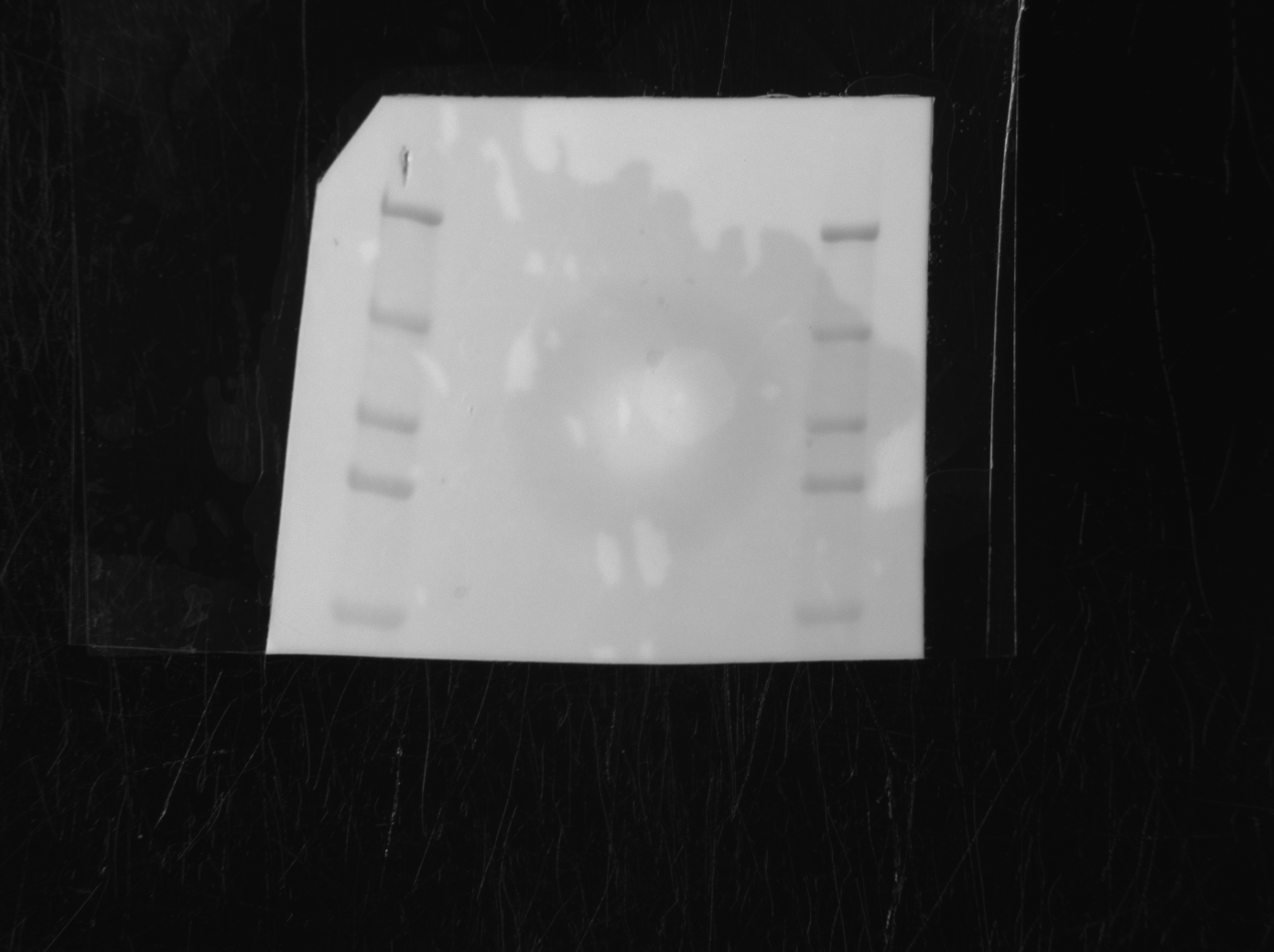

Supplement: Figure 5—source data 2. [file elife-85654-fig5-data2.zip › Figure 5-source data 2.tif]

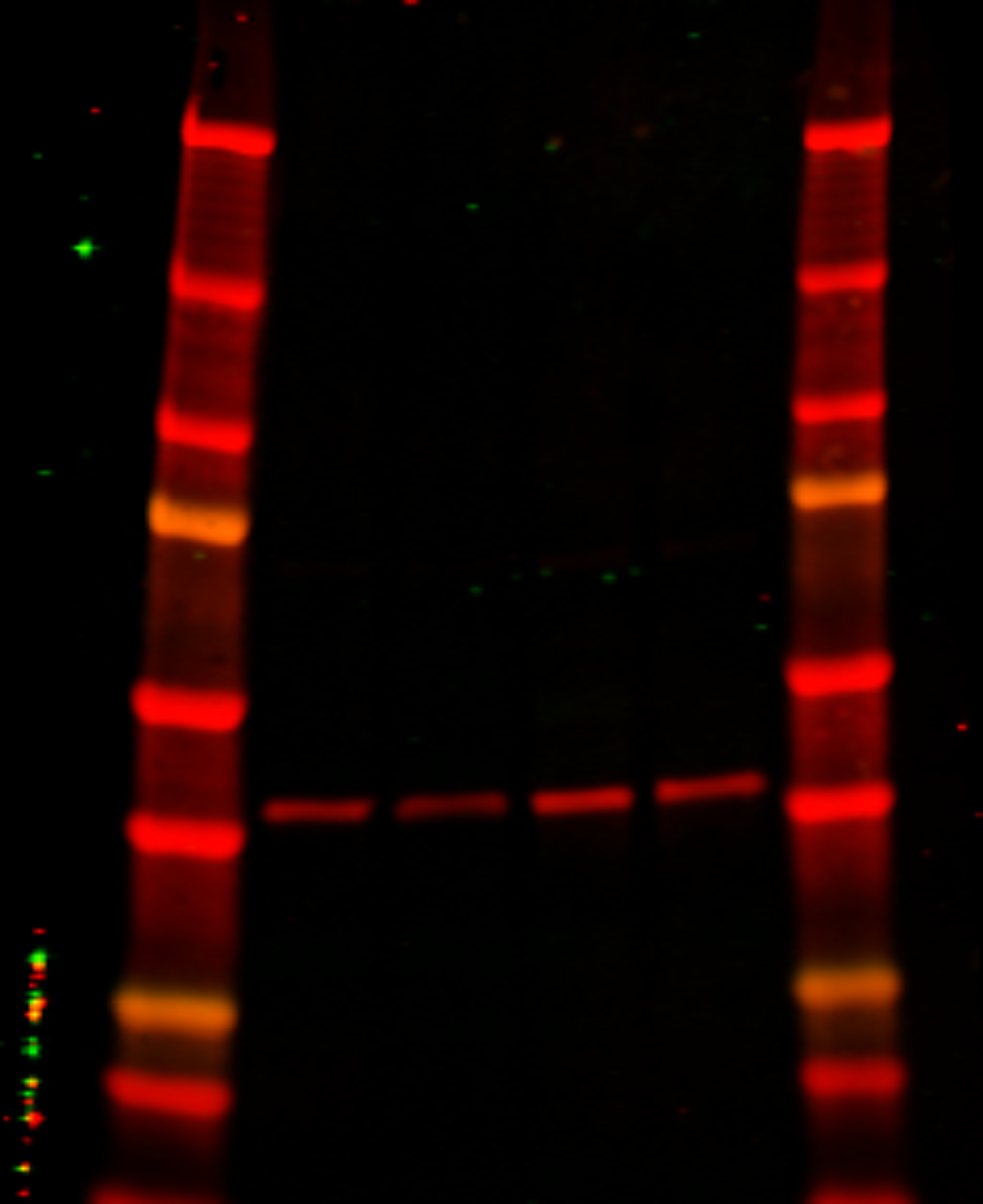

Supplement: Figure 5—source data 3. [file elife-85654-fig5-data3.zip › Figure 5-source data 3.tif]

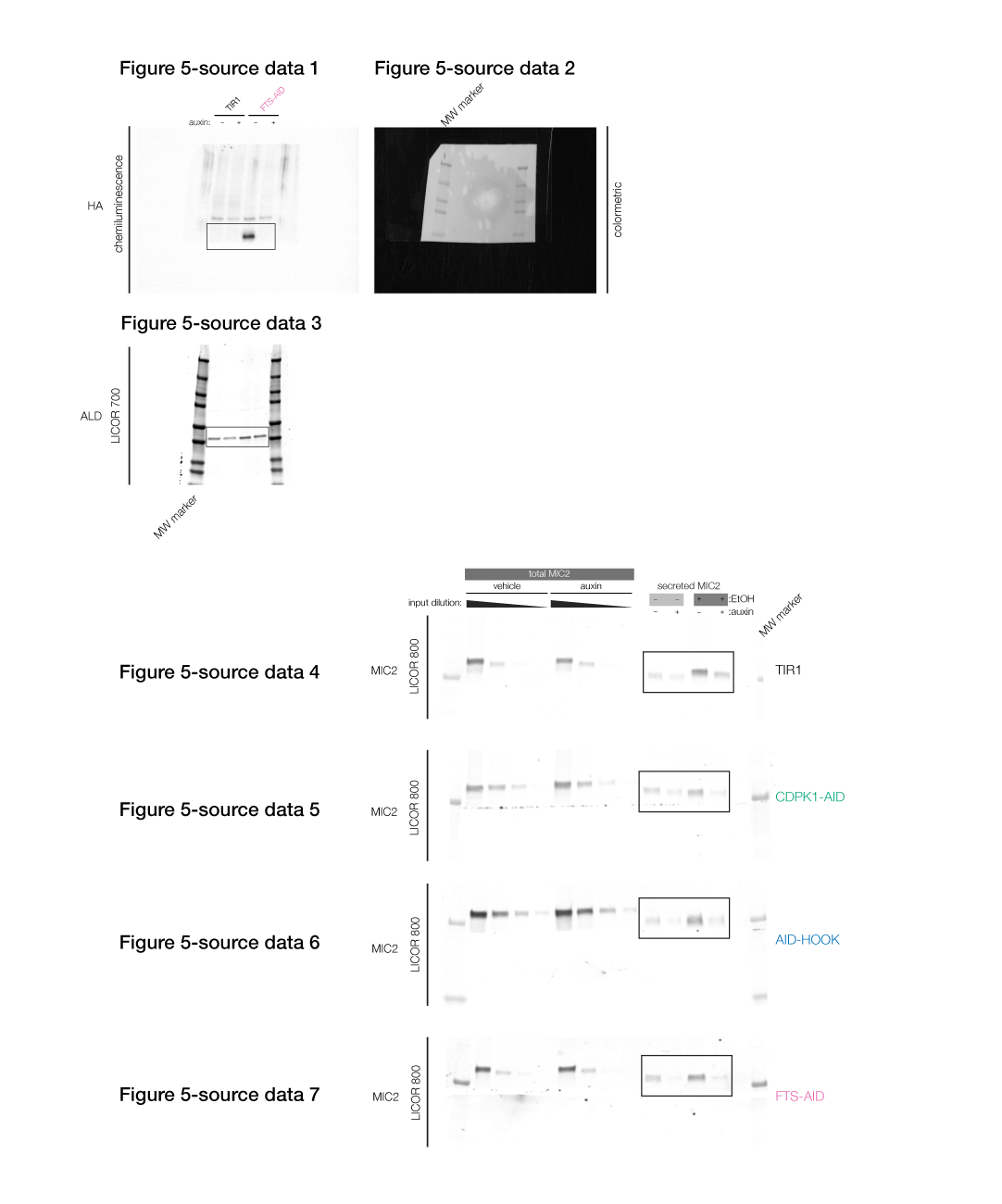

Supplement: Figure 5—source data 8. [file elife-85654-fig5-data8.zip › Figure 5-source data 8.tif]

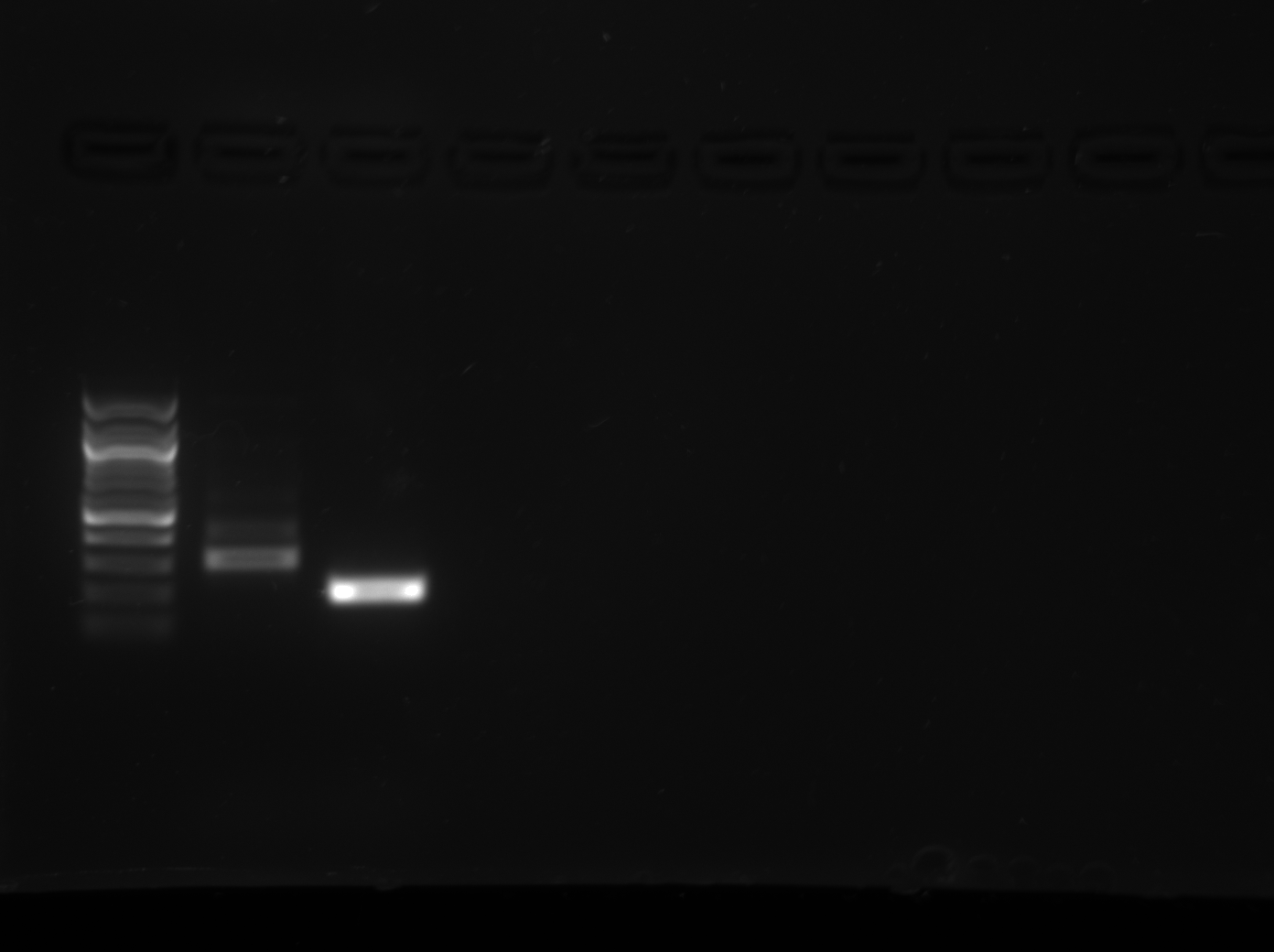

Supplement: Figure 5—figure supplement 1—source data 1. [file elife-85654-fig5-figsupp1-data1.zip › Figure 5-figure supplement 1-source data 1.tif]

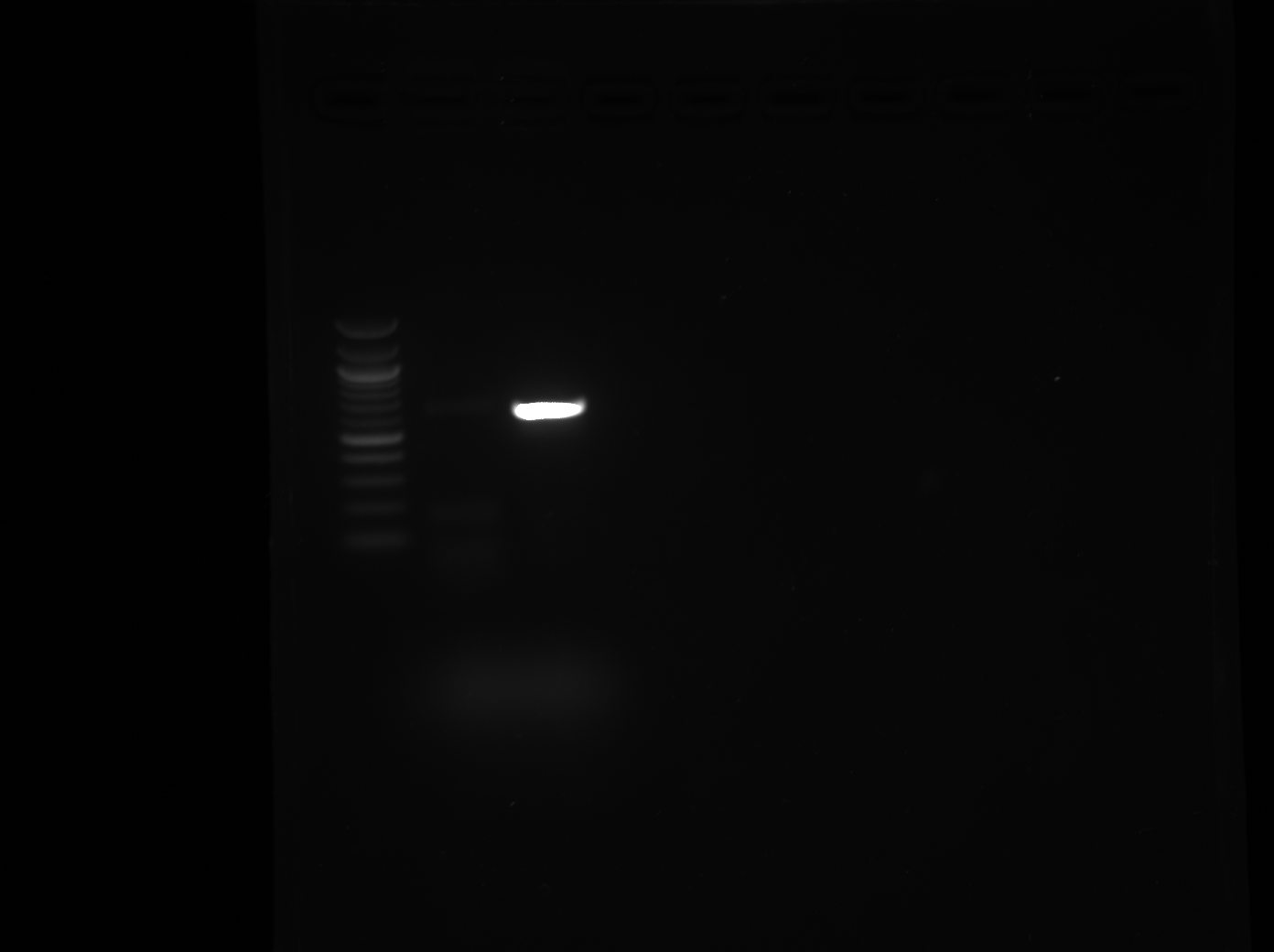

Supplement: Figure 5—figure supplement 1—source data 2. [file elife-85654-fig5-figsupp1-data2.zip › Figure 5-figure supplement 1-source data 2.tif]

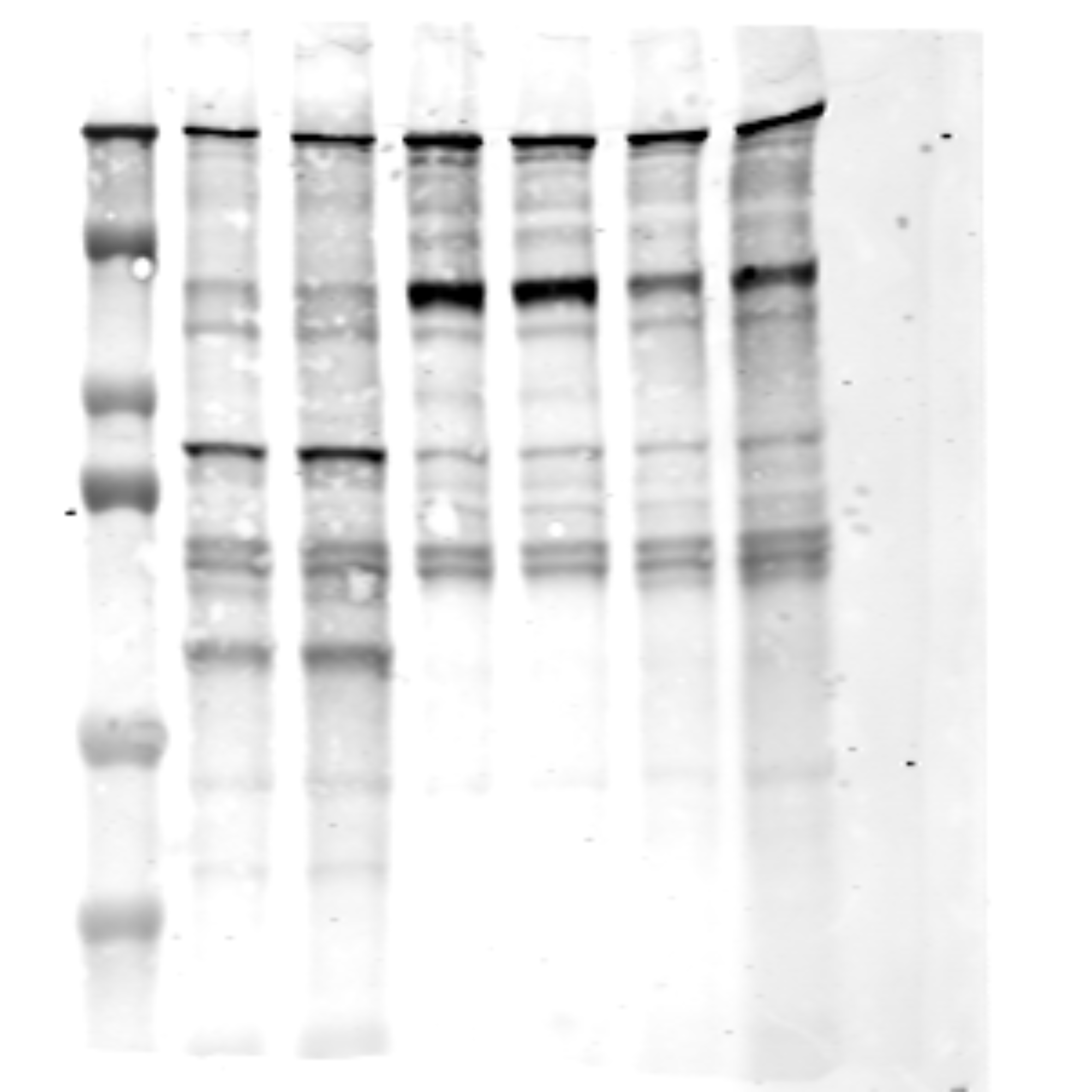

Supplement: Figure 5—figure supplement 1—source data 3. [file elife-85654-fig5-figsupp1-data3.zip › Figure 5-figure supplement 1-source data 3.tif]

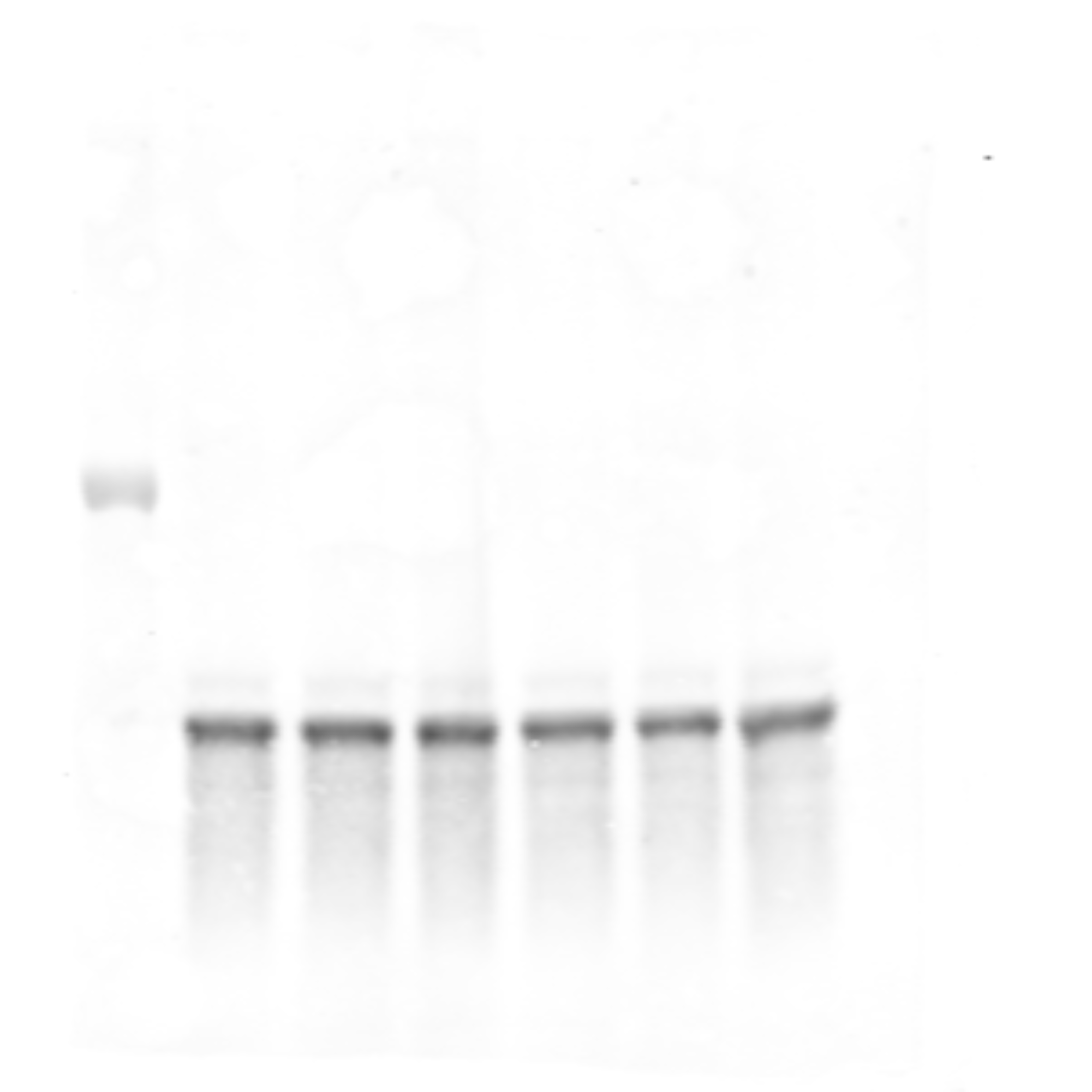

Supplement: Figure 5—figure supplement 1—source data 4. [file elife-85654-fig5-figsupp1-data4.zip › Figure 5-figure supplement 1-source data 4.tif]

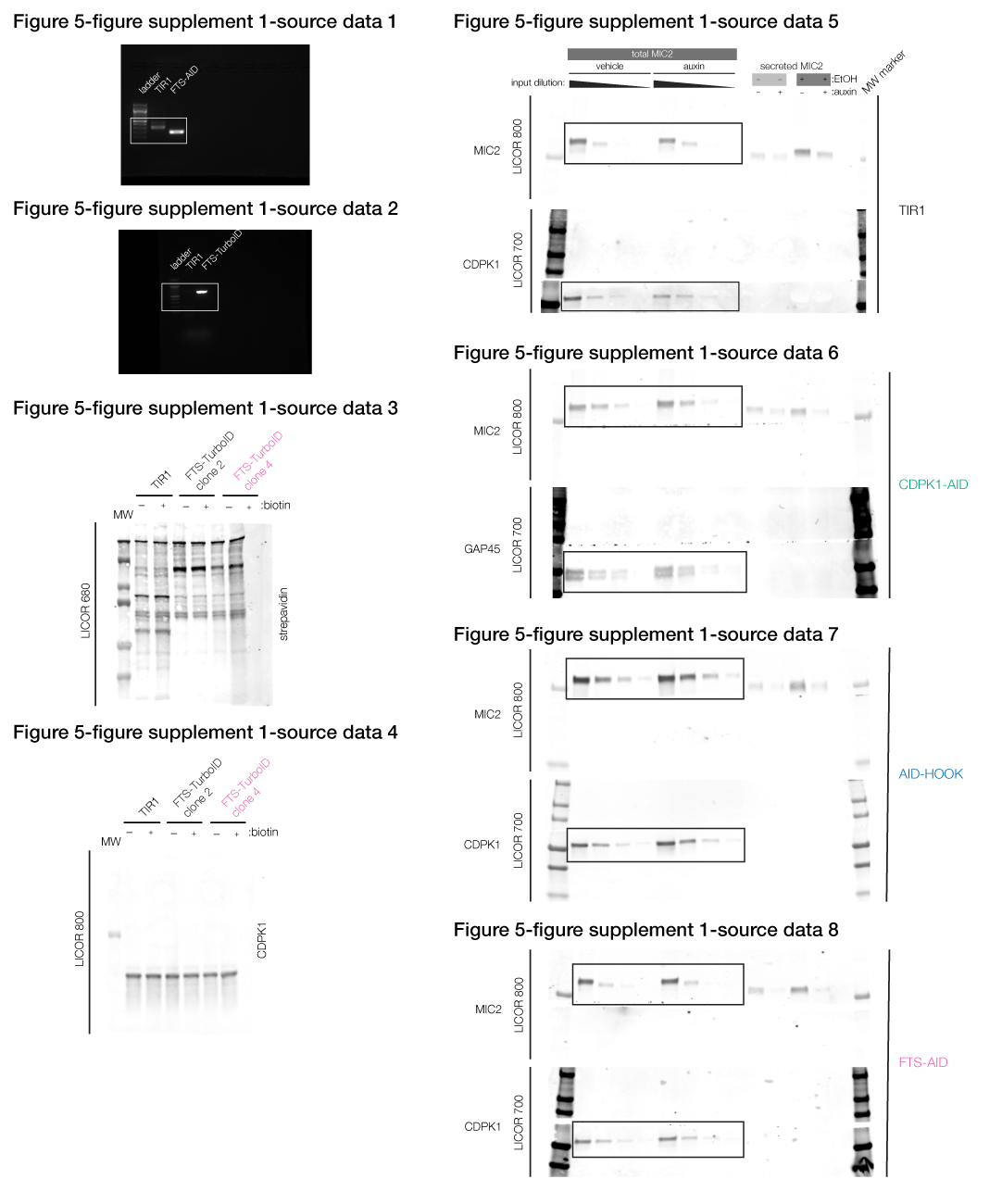

Supplement: Figure 5—figure supplement 1—source data 9. [file elife-85654-fig5-figsupp1-data9.zip › Figure 5-figure supplement 1-source data 9.tif]

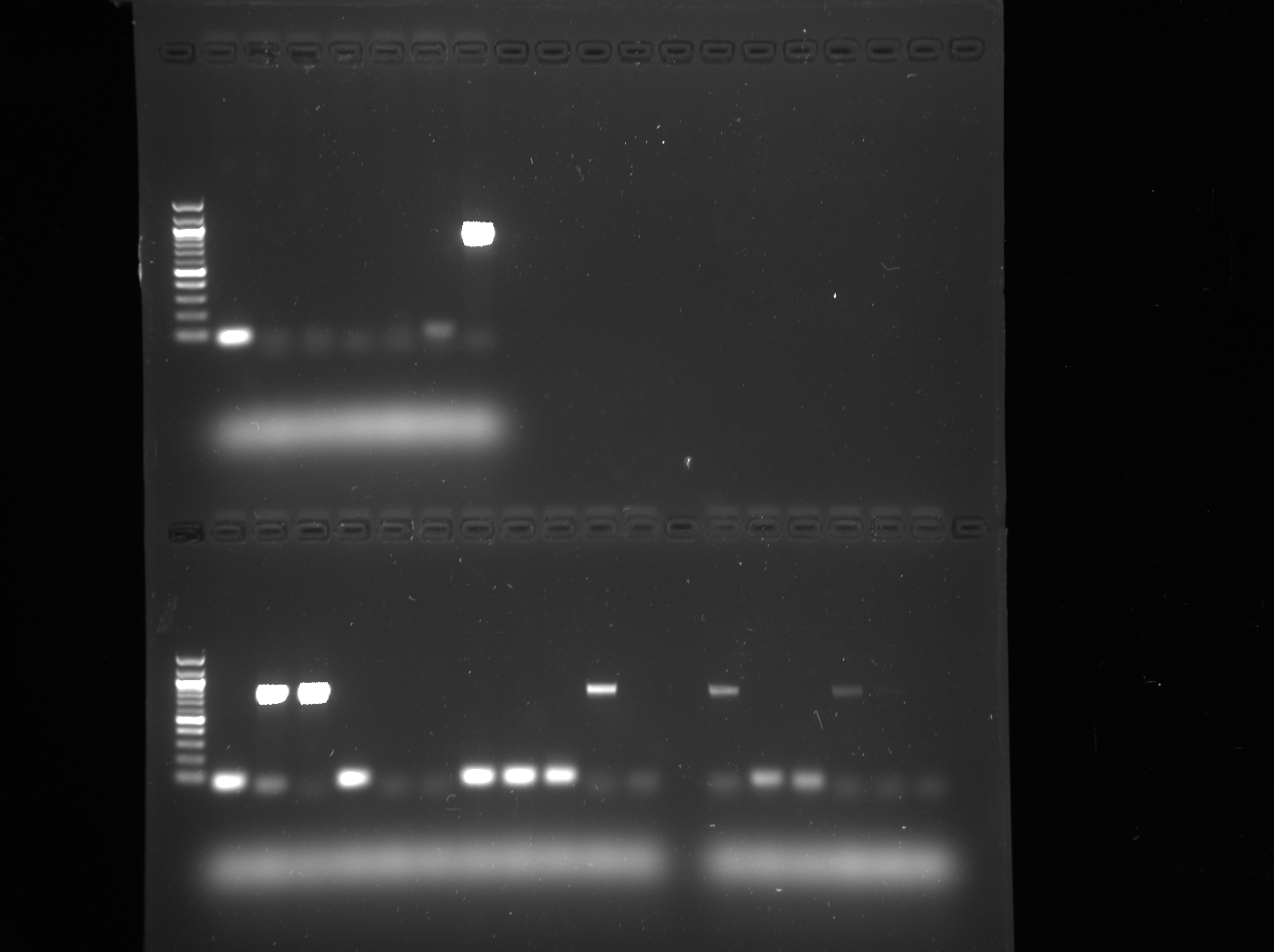

Supplement: Figure 6—figure supplement 1—source data 1. [file elife-85654-fig6-figsupp1-data1.zip › Figure 6-figure supplement 1-source data 1.tif]

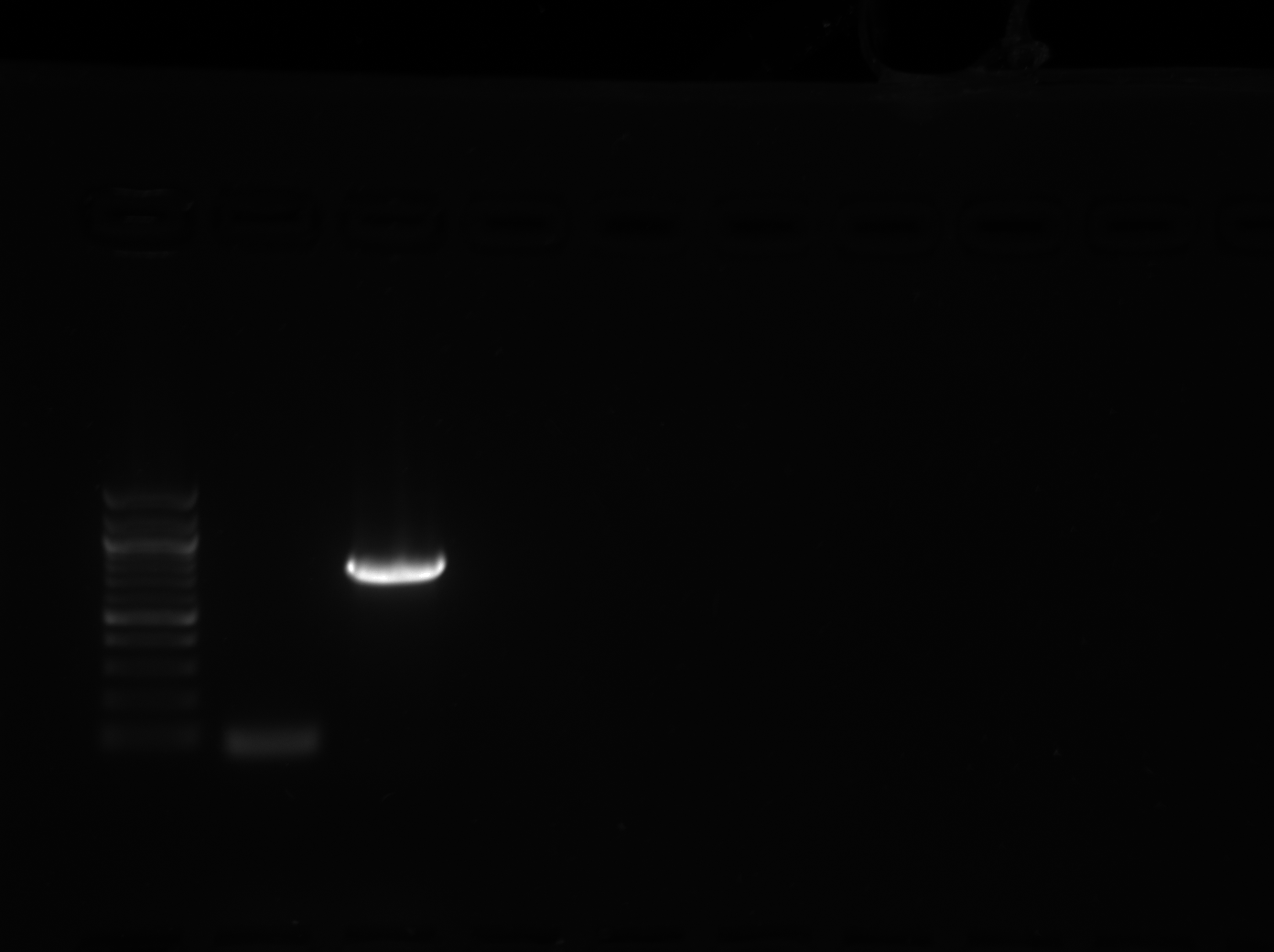

Supplement: Figure 6—figure supplement 1—source data 2. [file elife-85654-fig6-figsupp1-data2.zip › Figure 6-figure supplement 1-source data 2.tif]

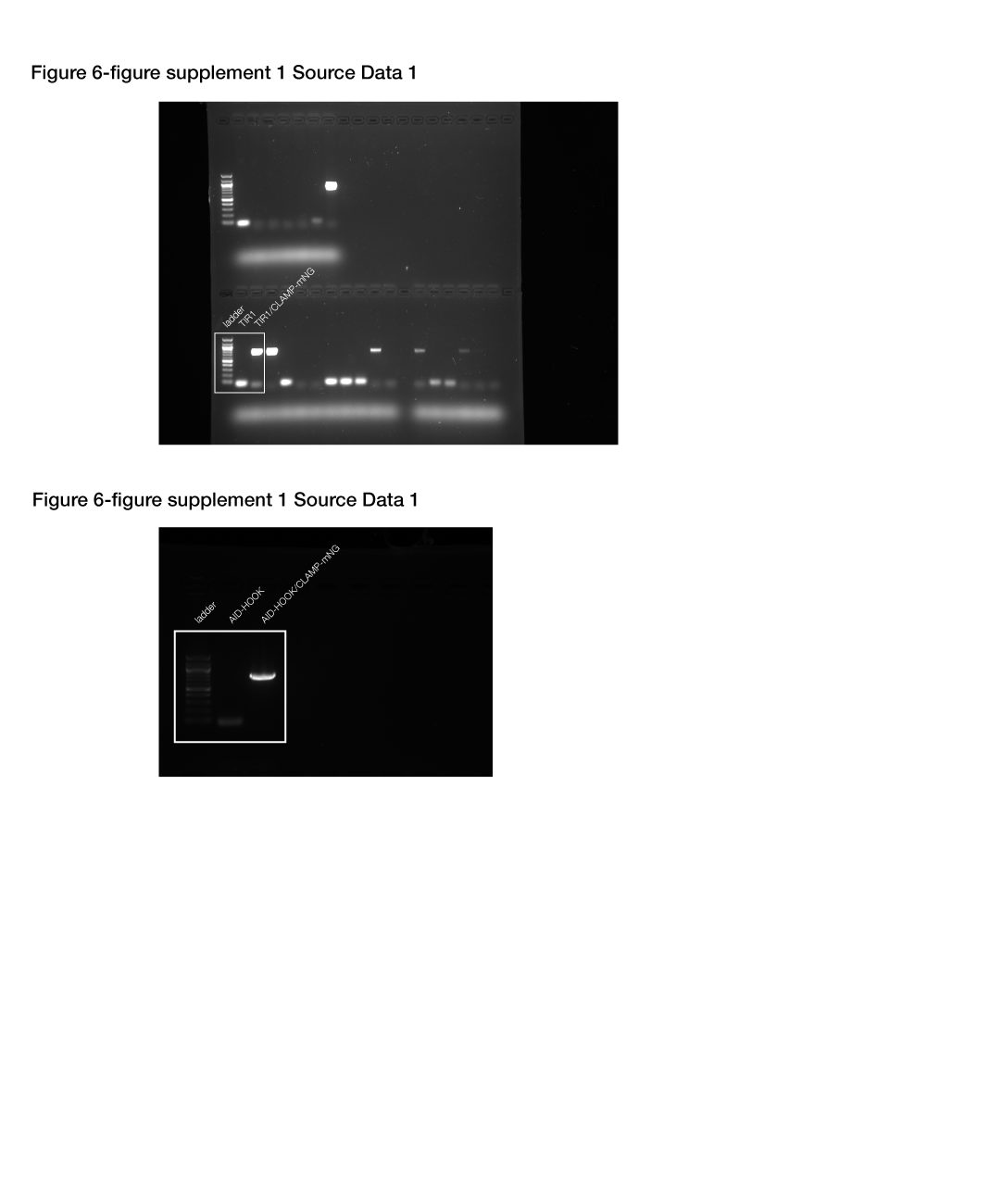

Supplement: Figure 6—figure supplement 1—source data 3. [file elife-85654-fig6-figsupp1-data3.zip › Figure 6-figure supplement 1-source data 3.tif]
